# Exploring serial crystallography for drug discovery

**DOI:** 10.1107/S2052252524006134

**Published:** 2024-07-29

**Authors:** A. Dunge, C. Phan, O. Uwangue, M. Bjelcic, J. Gunnarsson, G. Wehlander, H. Käck, G. Brändén

**Affiliations:** a Department of Chemistry and Molecular Biology University of Gothenburg Box 462 SE-405 30Gothenburg Sweden; b Mechanistic and Structural Biology, Discovery Sciences, BioPharmaceuticals R&D AstraZeneca Pepparedsleden 1 SE-431 83Gothenburg Sweden; c MAX IV Laboratory Lund University PO Box 118 SE-221 00Lund Sweden; d Discovery Biology, Discovery Sciences, BioPharmaceuticals R&D AstraZeneca Pepparedsleden 1 SE-431 83Gothenburg Sweden; University of Michigan, USA

**Keywords:** serial crystallography, microcrystals, drug discovery, temperature-dependent structural differences, fixed-target devices, room-temperature structures, soluble epoxide hydrolase

## Abstract

Structure-based drug design is highly dependent on the availability of structures of the protein of interest in complex with lead compounds. Ideally, this information can be used to guide the chemical optimization of a compound into a pharmaceutical drug candidate. A limitation of the main structural method used today – conventional X-ray crystallography – is that it only provides structural information about the protein complex in its frozen state. Serial crystallography is a relatively new approach that offers the possibility to study protein structures at room temperature (RT). Here, we explore the use of serial crystallography to determine the structures of the pharmaceutical target, soluble epoxide hydro­lase. We introduce a new method to screen for optimal microcrystallization conditions suitable for use in serial crystallography and present a number of RT ligand-bound structures of our target protein. From a comparison between the RT structural data and previously published cryo-temperature structures, we describe an example of a temperature-dependent difference in the ligand-binding mode and observe that flexible loops are better resolved at RT. Finally, we discuss the current limitations and potential future advances of serial crystallography for use within pharmaceutical drug discovery.

## Abstract 2

In this work, serial crystallography is applied to a drug discovery target and the room-temperature ligand-bound complexes are used to explore temperature-dependent structural differences.

## 1. Introduction

Structure-based drug design is a very well established concept and is integral to drug discovery since it can improve the speed and quality of pharmaceutical candidate compound discovery. Structural methods are commonly used throughout a drug-design project from early hit-finding to optimization of potency and properties for *in vivo* delivery (Maveyraud & Mourey, 2020 ▸). Thus, all major pharmaceutical companies and many biotech companies have access to structural biology capabilities. Successful structure-based drug design requires structure determination to high quality on a timescale matching that of the design cycle. Conventional macromolecular X-ray crystallography, performed by collecting X-ray diffraction data while continuously rotating a single protein crystal kept at cryogenic temperature, is highly streamlined and the work horse of structure-based drug design. It does, however, require a very robust crystallization protocol and includes the labour-intense step of harvesting crystals. Single-particle cryo-electron microscopy (cryo-EM) has gained much attention during the recent years as an attractive alternative to X-ray crystallography as it does not require that the protein of interest is prone to form well ordered crystals (Guaita *et al.*, 2022 ▸). However, both traditional cryo-crystallography and single-particle cryo-EM are limited in that they only provide structural information about the target protein in the frozen state.

Serial crystallography (SX) is a relatively new concept where X-ray diffraction data are collected on micrometre-sized crystals at room temperature (RT). The method was first developed for use at X-ray free-electron laser facilities, where the extreme intensity of the X-ray pulses required a new way of collecting diffraction data. To circumvent this problem, injection devices were developed that supply a stream of very small crystals across the X-ray beam such that each crystal that intercepts the X-rays gives rise to one diffraction image (Chapman *et al.*, 2011 ▸). Pioneering experiments established that, although the data recorded is built up of partial reflections from randomly oriented crystals, it is possible to obtain high-quality structural information using SX (Boutet *et al.*, 2012 ▸). Since then, the concept has been further developed at synchrotron facilities (Gati *et al.*, 2014 ▸; Martin-Garcia, 2021 ▸; Caramello & Royant, 2024 ▸), giving rise to the term serial synchrotron crystallography (SSX). There are now a number of purpose-built synchrotron beamlines that allow many more users to explore the method than what XFEL facilities can cater for, including T-REXX at PETRA III, Germany; ID29 at the ESRF, France; and MicroMAX at MAX IV, Sweden. The growth of the SX method has also led to a wide variety of sample delivery methods being developed. This includes different variants of jets, including the high-viscosity extruder injector, suitable for viscous samples such as membrane proteins crystallized in lipidic cubic phase (Weierstall *et al.*, 2014 ▸; Vakili *et al.*, 2023 ▸; Ghosh *et al.*, 2023 ▸). There is also a multitude of different fixed-target devices available where the crystals are dispensed onto a surface that is translated across the X-ray beam in a grid-like fashion. This includes highly specialized chips where the surrounding atmosphere of the crystal can be controlled (Roedig *et al.*, 2017 ▸), as well as simple X-ray-transparent membranes that are sandwiched around the crystals and mounted onto a standard crystallography pin (Roedig *et al.*, 2017 ▸, 2016 ▸; Owen *et al.*, 2017 ▸; Carrillo *et al.*, 2023 ▸; Mehrabi *et al.*, 2020 ▸), enabling data collection at any high-focus synchrotron beamline equipped with a fast translating goniometer. A large advantage of fixed-target devices is that sample consumption can typically be minimized compared with jets, a prerequisite for SX to be a realistic alternative for the majority of proteins.

A scientific area where SX has had great impact is within time-resolved structural studies, where the method has opened up unique possibilities previously out of reach for traditional cryo-crystallography (Brändén & Neutze, 2021 ▸). Of more general interest, it has been suggested that it may be important to study protein structures at RT as opposed to in their frozen state to reduce the risk of temperature artefacts. Several studies have made interesting observations of structural differences related to temperature (Fenwick *et al.*, 2014 ▸; Ebrahim *et al.*, 2022 ▸; Keedy *et al.*, 2018 ▸), including the loss of information about transient binding sites when working at cryogenic temperature (Fischer *et al.*, 2015 ▸). Moreover, RT structural information provided an increased understanding of the link between inhibitor potency and binding conformation in the study of a cancer target (Milano *et al.*, 2021 ▸). Recently, a crystallographic screen of 143 small-compound fragment binders targeting protein tyrosine phosphatase PTP1B was performed using rotational crystallography at RT (Skaist Mehlman *et al.*, 2023 ▸). By comparison with earlier cryo-crystallography data, it was shown that fewer ligands bound at RT, but also that unique binding poses were identified in the RT experiment, including interactions at new binding sites that had previously not been observed. Finally, SX has a potential advantage in that it may be possible to avoid the manual handling of crystals required in traditional cryo-crystallography, where the protein crystals are typically harvested and manipulated by hand. Future automation of crystal handling would be highly interesting, not the least for use in high-throughput drug discovery campaigns.

**Figure 1:**
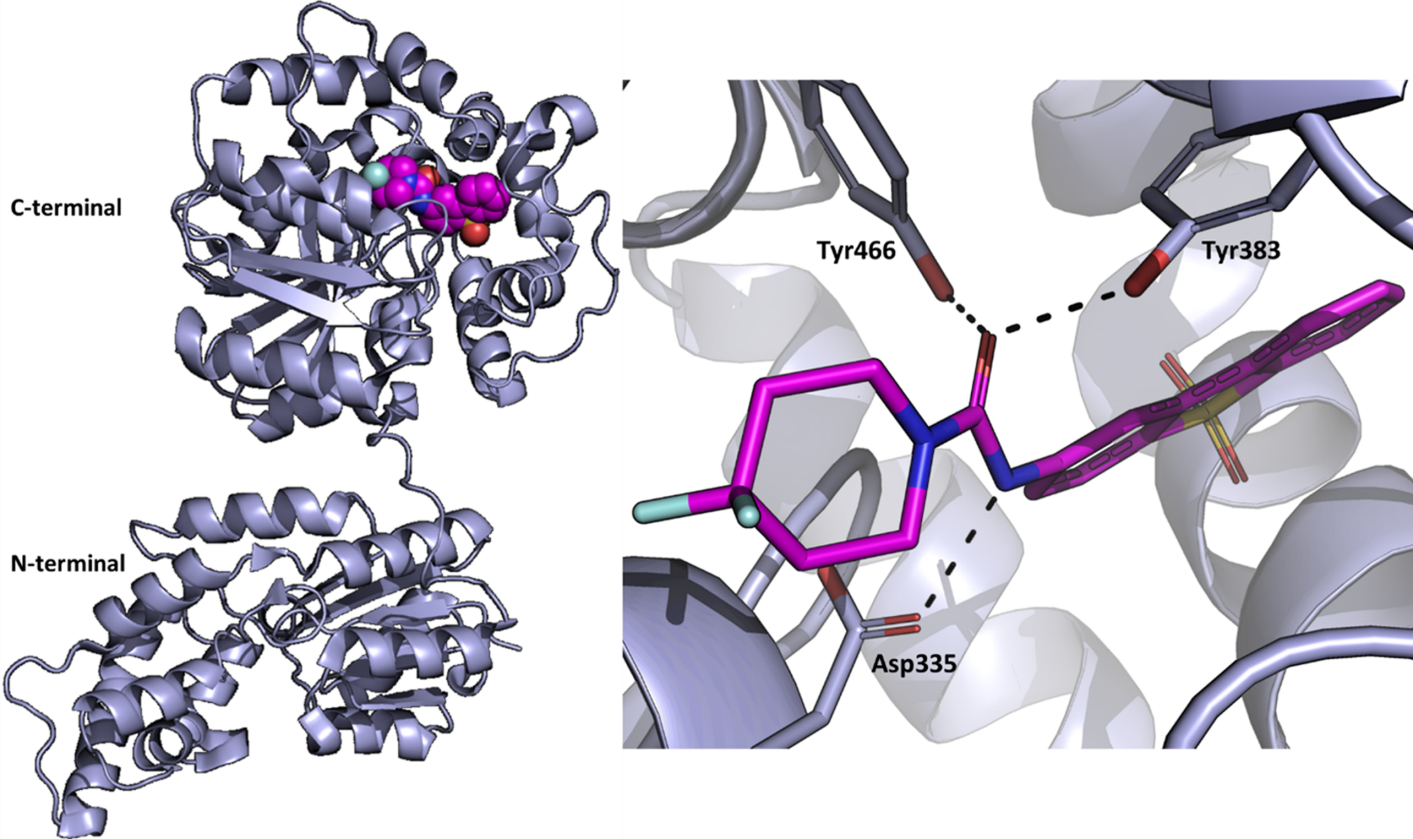
Structure of sEH. (Left) Structure of inhibitor-bound sEH solved at cryogenic temperature (PDB entry 5ake; Öster *et al.*, 2015 ▸). The active site with the bound compound 4 (purple) is located in the C-terminal domain. (Right) Zoom-in of the active site where the compound (purple) interacts with the catalytic triad (Asp335, Tyr383 and Tyr466).

To explore the utility of SX in a drug discovery setting, we selected a pharmaceutically relevant target – soluble epoxide hydro­lase (sEH) – as a model system. sEH is an enzyme that binds epoxides and converts them to their corresponding diols (Newman *et al.*, 2003 ▸). The human protein is 62.5 kDa in size and consists of a C-terminal and an N-terminal domain connected by a short proline-rich linker (Fig. 1 ▸). The C-terminal domain exhibits hydro­lase activity and the N-terminal domain is suggested to have phosphatase activity. Three residues within the C-terminal domain active site, ASP335, TYR383 and TYR466, make up the catalytic triad (Fig. 1 ▸). The inhibition of sEH can effectively maintain endogenous ep­oxy­eicosatrienoic acid (EET) levels and reduce di­hydroxy­eicosatrienoic acid (DHET) levels, thus providing therapeutic potential for cardiovascular, central nervous system and metabolic diseases (Imig & Hammock, 2009 ▸; Chiamvimonvat *et al.*, 2007 ▸; Sun *et al.*, 2022 ▸; Shen & Hammock, 2012 ▸; Hashimoto, 2019 ▸; Vázquez *et al.*, 2023 ▸). Structures of sEH in complex with a large number of inhibitors have been solved previously using conventional cryo-crystallography (Öster *et al.*, 2015 ▸). Thus, we set out to generate a set of RT protein–inhibitor complexes to compare with those previously solved and explore whether the use of SX would provide additional structural information of interest in a drug discovery setting.

## 2. Methods

### 2.1. Protein production and purification

As full length sEH has a disordered C-terminal, a C-terminally truncated construct of sEH(1–548) was expressed in *Spodoptera frugiperda* (*SF*9) insect cells as previously described (Öster *et al.*, 2015 ▸). 100 ml of cells were used for each purification following the published protocol with the exception that two interconnected 5 ml HisTrap FF Crude (Cytivia) columns were used instead of one to avoid saturation of the column. The purified protein was concentrated to 15–20 mg ml^−1^ and stored in a buffer containing 20 m*M* Tris–HCl pH 8, 150 m*M* NaCl, 1 m*M* TCEP and 10% glycerol.

### 2.2. Crystallization

A concentrated seed solution was generated by growing larger crystals of sEH, 100–1000 µm in size, following a previously established protocol (Öster *et al.*, 2015 ▸). In brief, vapour diffusion crystallizations were set up using a protein concentration of 15–20 mg ml^−1^ and a 1:1 ratio of protein:precipitant solution in 24-well sitting drop plates (Cryschem M Plate, Hampton research) with a well solution containing 32–38% PEG3350, 0.1 *M* LiSO_4_, 0.1 *M* Tris pH 8.5 and a drop volume of 5–10 µl. The drops were streak-seeded using crystal seeds previously obtained using this protocol. Drops containing well formed crystals were transferred into a 2 ml Eppendorf tube, typically resulting in a volume of ∼100 µl. Two microseed beads (Molecular Dimensions) were added to the tube and the crystals were crushed by vortexing the sample for 3 × 5 min, or until no large fragments of crystals were visible in an optical microscope. This seed production protocol was inspired by a previously published method to produce seeds for microcrystallization (Dods *et al.*, 2017 ▸).

Optimization of batch crystallization conditions was carried out in a micro-batch setting using sitting-drop plates, referred to as hybrid crystallization. This was achieved by composing the well solution such that the concentration of the individual components corresponds to the concentration in the crystallization drop after the addition of protein. A crystallization screen was carried out in this fashion by varying the seed concentration and seed volume. In addition, the concentrations of protein, PEG3350, Li_2_SO_4_ and Tris–HCl (pH 8.5) were optimized, as well as the ratio of protein to precipitant solution (see Table S1 of the supporting information for details).

**Figure 2:**
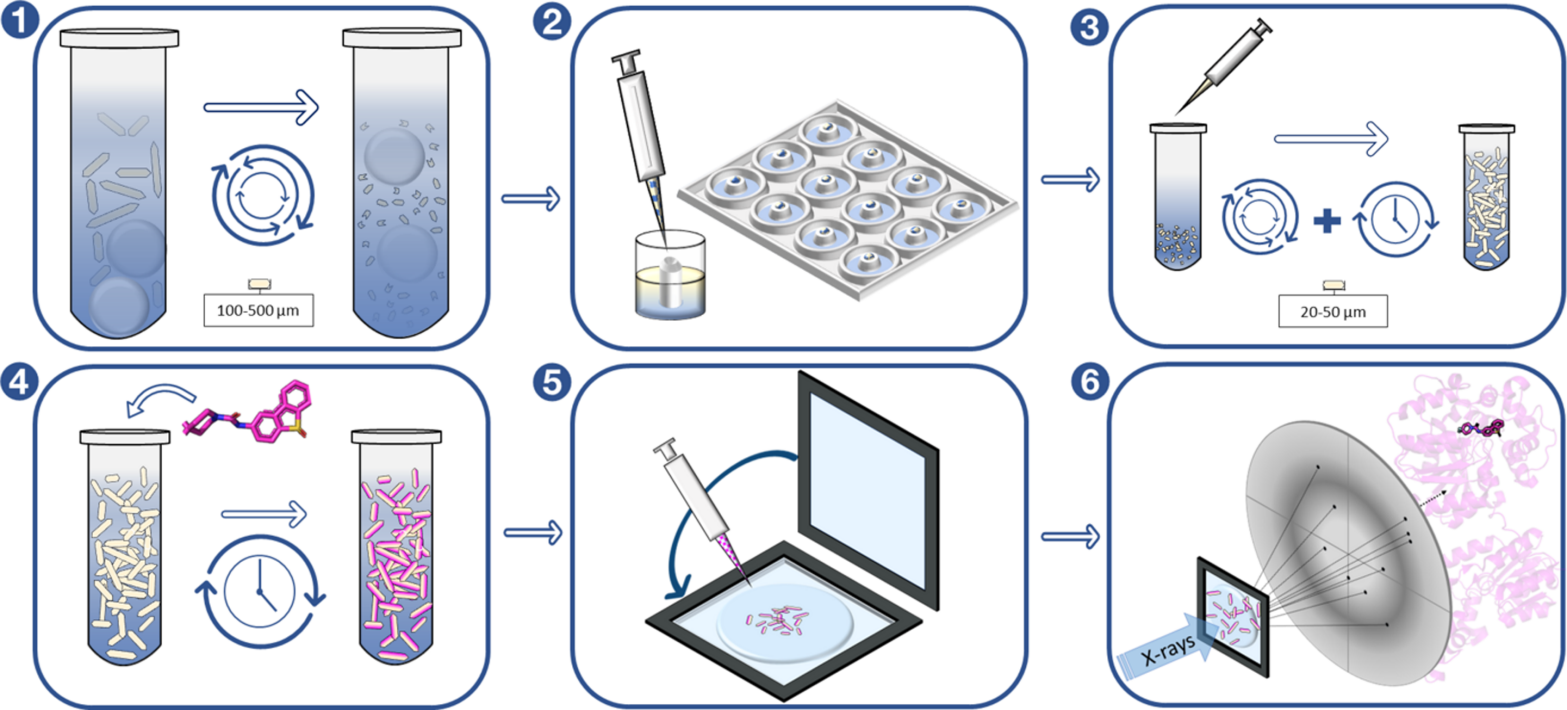
Microcrystallization and data collection workflow. Panel 1: production of crystal seeds from macro crystals using seed beads and vortexing. Panel 2: hybrid crystallization used for screening to find optimal batch crystallization conditions. Panel 3: batch crystallization with seeding and vortexing to induce nucleation. Panel 4: soaking of compound into the microcrystals. Panel 5: dispensing of soaked crystals onto the chip and sealing. Panel 6: fixed-target raster grid-scan data collection on ligand-soaked crystals at a synchrotron source.

Optimized microcrystals for SX data collection were produced as follows (Fig. 2 ▸). A crystallization solution was prepared by mixing 10%(*v*/*v*) seed solution into a precipitant solution containing 34% PEG3350, 0.1 *M* Tris–HCl (pH 8.5) and 0.1 *M* Li_2_SO_4_. Protein at 14 mg ml^−1^ was then mixed with the crystallization solution at a 1:4 protein:precipitant/seed solution ratio in a 0.5 ml Eppendorf tube and the solution was vortexed. The total microcrystallization volume was typically 50–100 µl. The sample was kept upright to allow the crystals to sediment to the bottom of the tube and was then stored at 20°C.

Each compound was dissolved in 99.5% DMSO to make up a stock solution with a concentration of 500 m*M* used to prepare the soaking solution. The soaking solution was prepared by diluting the dissolved compound in a buffer [43.5%(*w*/*v*) PEG3350, 0.1 *M* Tris–HCl (pH 8.5) and 0.1 *M* Li_2_SO_4_], spun down to remove any precipitated ligand and mixed into the crystal slurry at a final concentration of 10–50 m*M*. Microcrystals were incubated with the compound for 2–72 h before data collection.

### 2.3. Data collection and processing

All data collection was performed at the MX beamline BioMAX of MAX IV Laboratory using a fixed-target SX setup (Shilova *et al.*, 2020 ▸). To increase the crystal hit-rate, the crystals were concentrated by centrifugation for 5–30 s at 1500*g*. Next, 1.8–2 µl of the crystal solution was sandwiched between two Silson membranes (SiRN-5.0-200-2.5-1000) and manually mounted onto the goniometer using a reusable pin. Room-temperature diffraction data were collected on a Dectris Eiger 16M hybrid pixel detector by raster grid scanning using a 20 × 20 µm X-ray beam with 100% transmission and an exposure time of 11 ms.

The datasets varied in size between 50 000 and 100 000 images. Hit finding, indexing and integration were achieved using *indexamajig* in the *CrystFEL* suite (White *et al.*, 2012 ▸; White, 2019 ▸). *Partialator* was used for merging and scaling of the data. The high-resolution limit of each dataset was determined based on a CC_1/2_ value of at least 30 and an *I*/σ(*I*) value of at least 0.7, unless the CC_1/2_ parameter indicated that the data in a lower-resolution shell were problematic, in which case a lower resolution cut-off was selected. The structures were solved by molecular replacement in the *ccp4i* suite (Agirre *et al.*, 2023 ▸) using a structure of sEH solved by conventional crystallography at cryo-temperature (cryo-T) as a search model (PDB entry 9exm). The model was subsequently refined using *BUSTER* (Bricogne *et al.*, 2017 ▸), while *Coot* (Emsley & Cowtan, 2004 ▸) was used for real-space rebuilding and refinement. Ligand restraints were generated with either *writedict* or *Grade* (Smart *et al.*, 2011 ▸), and *AFITT* (Open Eye Scientific Software; Wlodek *et al.*, 2006 ▸). The software *PyMOL* (Schrodinger, 2015 ▸) was used to produce the figures. For data processing and refinement statistics, see Table 1 ▸.

## 3. Results and discussion

### 3.1. Crystallization and data collection

#### 3.1.1. Development of a new crystallization work-flow for SX

A key factor for a successful SX study is the ability to generate a sample of microcrystals homogeneous in size, of high quality and in sufficient quantity (Dods *et al.*, 2017 ▸). Thus, a robust crystallization protocol is required. For the purpose of generating the quantities needed, batch crystallization has typically been used for growing microcrystals (Beale *et al.*, 2019 ▸). A severe limitation when using batch crystallization, however, is that it typically requires very large volumes of protein already at the screening stage. Another disadvantage is that visual inspection of crystallization progress is difficult. Our so-called hybrid crystallization approach, described below, presents a solution to both these problems.

Initial microcrystallization trials of sEH using vapour diffusion in sitting-drop plates with the addition of seeds showed that reducing the protein concentration or increasing the concentrations of Li_2_SO_4_ and PEG3350 decreased the size of the crystals. In this study, we aimed for a crystal size of 20–40 µm in length as this was expected to give a good balance between high diffraction and a low number of overlapping crystals on the grids.

The main issue with the initial sitting-drop experiments was the low number of crystals generated in each drop. This is problematic as a low crystal density gives a low hit-rate (*i.e.* a low number of recorded diffraction images). It was possible to increase the number of crystals by several orders of magnitude by adding seed solution to the drop; however, this also induced the formation of precipitate. The problem was in part overcome by screening each new seed stock and testing dilutions between 1:16 and 1:256 of the seed stock in 43%(*w*/*v*) PEG3350 0.1 *M* Tris–HCl (pH 8.5) and Li_2_SO_4_, where 10%(*v*/*v*) seed stock was added to the precipitant solution in each case, to ensure an optimal number of crystallization nuclei. If available, using a device such as a hemocytometer for crystal counting (Boudes *et al.*, 2017 ▸) would likely have improved the reproducibility of this step, as has been shown by others (Shoeman *et al.*, 2023 ▸). Crystals could be detected after 1 h at 20°C. To reduce the speed of crystal formation and thereby potentially improve the crystal packing, crystallization experiments were also performed at 12 and 4°C. The lower temperatures reduced the speed of crystal growth but also induced the formation of aggregates, thus all further setups were carried out at 20°C.

Batch crystallization is typically the preferred method to produce large amounts of microcrystals. However, in the case of sEH, optimal crystallization conditions for vapour diffusion experiments with drop sizes of 1–3 µl did not translate well into batch conditions with final volumes of 50–100 µl. Since it is not feasible to screen a large variety of conditions in batch due to the high protein consumption, we developed an alternative method that we refer to as hybrid crystallization.

**Figure 3:**
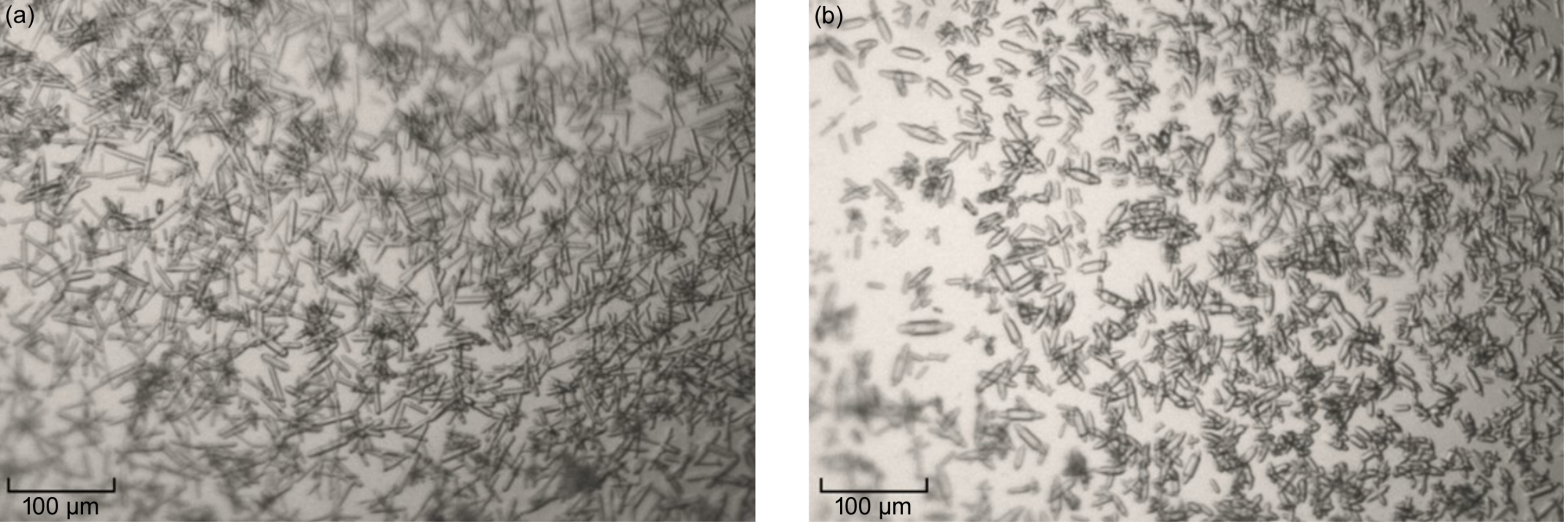
Microcrystals of sEH. The effect of using different protein:precipitant/seed solution ratios during screening for optimal conditions using the hybrid crystallization method is shown. (*a*) A protein:precipitant/seed solution ratio of 1 is used. (*b*) A protein:precipitant/seed solution ratio of 2 is used. A ratio of 2 gives a lower number of crystals but a more homogeneous sample with fewer overlapping crystals.

Hybrid crystallization is done in regular sitting-drop plates where the solution in the reservoir well is a mixture of the precipitant solution and the protein buffer in the same ratio as that used between the precipitant solution and protein in the drop. Therefore, the drop size will not change over the course of the experiment as in a regular vapour-diffusion setup. Instead, the experiment is very similar to a batch setup, but without risking that the small-volume drop dries out. The main advantage of this micro-batch setup is that sample consumption can be kept very low while screening for optimal crystallization conditions. Moreover, the hybrid crystallization approach allows the screening to be performed in plates which facilitates visual inspection during the course of the experiment. Finally, the hybrid crystallization method can take advantage of automated dispensing to make it less labour intensive. Using a drop volume of 4–10 µl and a reservoir volume of 500 µl, a large number of conditions were tested. It was found that the protein to precipitant/seed solution ratio influenced the thickness of the crystals (Fig. 3 ▸) and thereby the resolution obtained. If the width of a single crystal was below 10 µm then the resolution in most cases would be 3 Å or worse. The optimal conditions identified were a precipitant solution of 34% PEG3350, 0.1 *M* Tris–HCl (pH 8.5) and 0.1 *M* Li_2_SO_4_ with the addition of 10%(*v*/*v*) seeds in combination with a protein:precipitant/seed solution ratio of 1:4. This condition could be successfully translated to larger-scale batch setups of 50–100 µl in tubes for microcrystallization of sEH. Microcrystals of sEH were sensitive to transportation, which resulted in crystals either melting or aggregating in transit. This was possibly due to temperature variations during shipping, and the best diffraction was obtained from crystals produced on-site at the synchrotron facility. However, to make SX viable for drug discovery applications, where the optimal working model relies on crystals being produced in-house and then sent to a synchrotron for remote data collection, it is crucial to develop a logistic chain that can maintain an environment where crystals can be shipped in solution or dispensed onto a chip.

#### 3.1.2. Ligand introduction

For this study, we selected a subset of ligands that had previously been structurally characterized in complex with sEH using conventional cryo-crystallography (Öster *et al.)* in order to be able to make a direct comparison of the resulting structures. The selected ligands range from small weakly binding compounds (*i.e.* fragments) to larger potent inhibitors of sEH (Table S2 and Fig. S1 of the supporting information). Crystals of ligand-bound protein were prepared by soaking the crystals in a solution of high-concentration compound (10–50 m*M* final concentration) containing 43.5%(*w*/*v*) PEG3350, 0.1 *M* Tris–HCl (pH 8.5) and 0.1 *M* Li_2_SO_4_ for a duration of 2 h or longer. We noted that several of the ligands were not fully solubilized under these conditions. Although it is quite common in soaking experiments that the ligand precipitates, it is seldom detrimental in conventional cryo-crystallography where the crystal is fished out of the drop prior to data collection. However, in fixed-target SX experiments, the crystal solution is added to a chip and thus ligand precipitate may result in high background and powder diffraction which negatively affect the data quality. Thus, more care must be taken when working with poorly soluble ligands in SX experiments. To reduce the problem caused by ligand precipitation, the ligand solution was centrifuged before addition to the crystal slurry.

#### 3.1.3. Data collection and processing

RT diffraction data were collected at the BioMAX beamline of MAX IV from crystals soaked with seven different compounds. A number of different fixed-target chips were explored for the SX data collection where the best results were achieved using the Silson membranes. We found that an X-ray flux of at least 1 × 10^12^ photons s^−1^ with 100% transmission were required for good diffraction. Each chip gave rise to 15 000–20 000 images on average and required 15–20 min to assemble and collect data from. Depending on the hit-rate and the number of diffraction images aimed at, a complete dataset was compiled out of data collected from 4–6 chips. Thus, each dataset was acquired in about 1–2 h. Interestingly, the fraction of indexed images varies between 6 and 69%, with compound 2 being the outlier on the low side. The main reason for this large variation lies in how the data collection grids are drawn on the chips (*i.e.* how carefully areas without crystals or of low crystal density were avoided). Other reasons may be variations in crystal density of the crystal batch, and that some compounds cause precipitate to form in the crystalline sample, thus lowering the indexing rate. As further discussed in Section 3.3, the high redundancy of the resulting data (up to 1700, see Table 1 ▸) suggests that the number of images could be drastically reduced. Combined optimization of the crystal density and data collection strategy could thus significantly reduce the data collection time and thereby make SX a more feasible option from a synchrotron usage perspective. Data collection, processing and refinement statistics are presented in Table 1 ▸.

### 3.2. Room-temperature structures of sEH and comparison with cryo-temperature structures

In this study, we present seven structures solved by SX at resolutions ranging from 2.1 to 2.5 Å resulting from sEH microcrystals soaked with different ligands. In addition, we solved an RT structure in the absence of a ligand (the so-called apo structure) to be used for comparison with the ligated complexes. Structures in complex with the same ligands previously solved at cryo-T were reported to be of similar resolutions [1.9–2.5 Å (Öster *et al.*, 2015 ▸)]. The microcrystals belonged to the space group *P*6_5_22 which is the same as previously published sEH data.

Overall, the structures solved at RT agree well with the previously reported cryo-crystallography structures with a root-mean-square deviation of the Cα positions of 0.3 Å between the apo structures solved at cryo-T and RT [PDB entries 5ahx (Öster *et al.*, 2015 ▸) and 8qvm, respectively]. Interestingly, the apo structure determined by SX at RT harbours an extended-differences density in the active site, which is not present in the apo structure determined by cryo-crystallography. The shape of the density agrees well with a bound PEG fragment, and was modelled as such (Fig. 5).

### 3.3. Ligand binding

**Figure 4:**
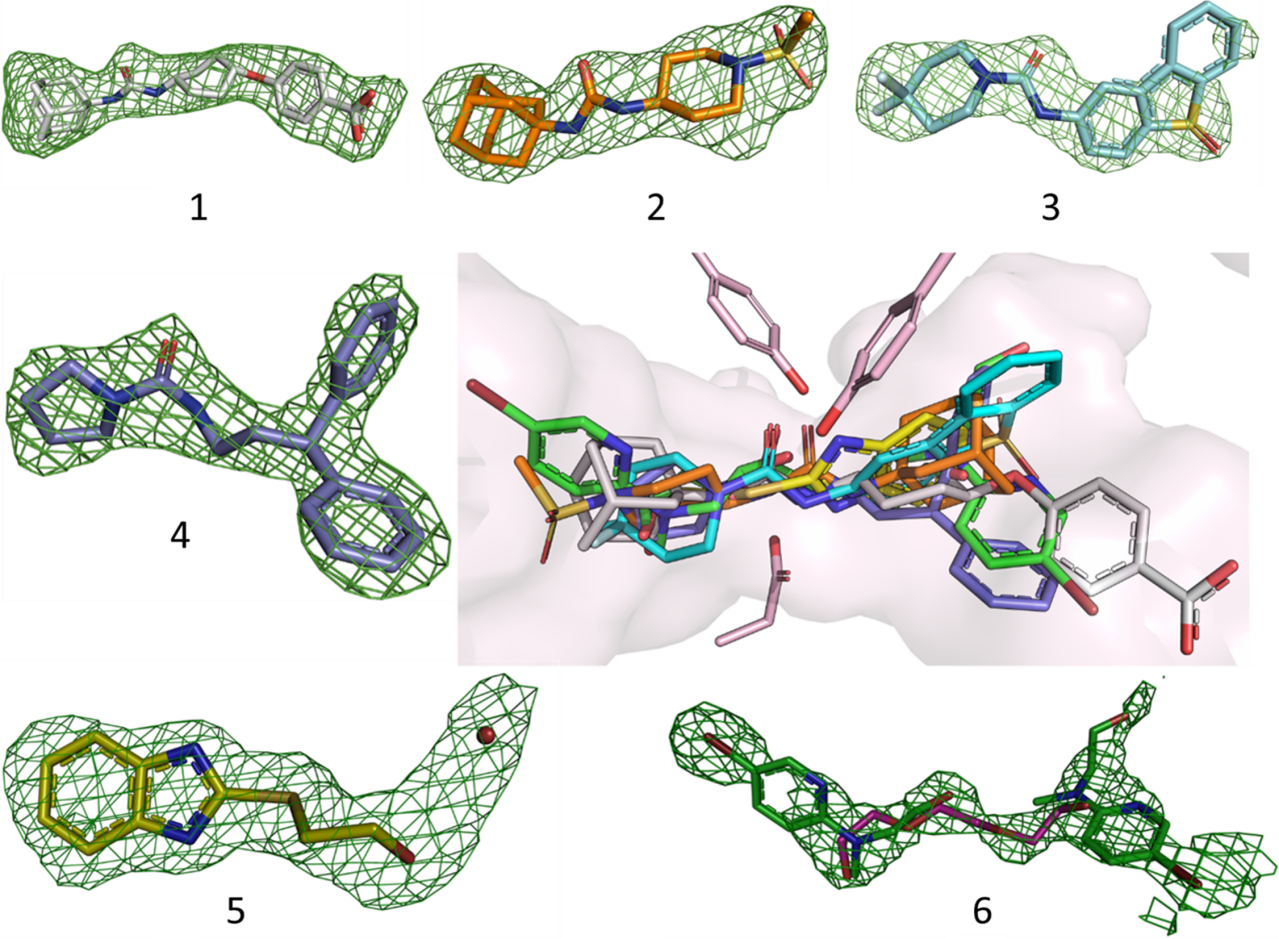
RT structures of sEH in complex with compounds. The outer images show the different compounds that form complexes numbered according to Table S2. The *F*_o_*F*_c_ omit difference electron density map (green) is contoured at +3.0σ in each case. The central panel displays an overlay of the seven RT sEH complex structures zoomed-in on the active site.

All the ligands with the exception of compound 7 could be identified in the active site binding pocket (Fig. 4 ▸). The large, potent ligands (compounds 1–4) have distinct chemical features, such as the adamatyl moiety of compounds 1 and 2, and the tetrahedral sulfur atoms of compounds 2 and 3 (Fig. S1) which are readily recognizable and could be unambiguously modelled in the electron-density maps.

Among the ligands, three compounds have a molecular weight below 300 Da and can be classified as fragments. Typically, fragments are weak binders and are therefore often associated with ambiguous electron density features due to partial occupancy (Schiebel *et al.*, 2016 ▸). In addition, it is not uncommon to find several copies of the same fragment bound in a structure when performing fragment screening due to the high compound concentrations used. Thus, maps from fragment-soaking experiments can be more difficult to interpret than those generated with more potent compounds.

The electron density corresponding to compound 5 has a shape which clearly fits the bi-aryl ring with the adjacent sulfur atom, displaying a stronger peak than the surrounding atoms (Fig. S2). An alternative effort to explain the density as a bound PEG fragment, based on the apo structure, gave an unconvincing result. There is a diffuse electron density feature extending from the hydroxyl group of compound 5 which is modelled as a water molecule.

The electron density map calculated from data collected on crystals soaked with compound 6 is more complicated to interpret and different options were explored (Fig. S3). The omit-map electron density extends over a large part of the binding pocket and cannot be accounted for by a single PEG fragment bound in the central position as in the apo structure. Also, attempts to fit the density with two PEG fragments do not produce a convincing result. Instead, as compound 6 contains a bromide atom, the presence of a strong peak in the omit-map electron density (‘left-hand side’ of the pocket) clearly identifies the position of one compound 6 molecule bound (although not at full occupancy). This binding pose is identical to that found in the cryo-T structure. Moreover, the omit-map density also suggests the binding of a second copy of compound 6 in the active site (‘right-hand side’ of the pocket), again similar to what is found in the previously solved cryo-T structure. The density in this part of the pocket is of lower quality and it is more difficult to obtain a completely satisfactory result. Overall, the best fit of the electron density is obtained with a model that includes a 50% partially occupied PEG fragment in the same position as in the apo structure, in combination with two copies of compound 6 at 50% occupancy (Fig. S3).

**Figure 5:**
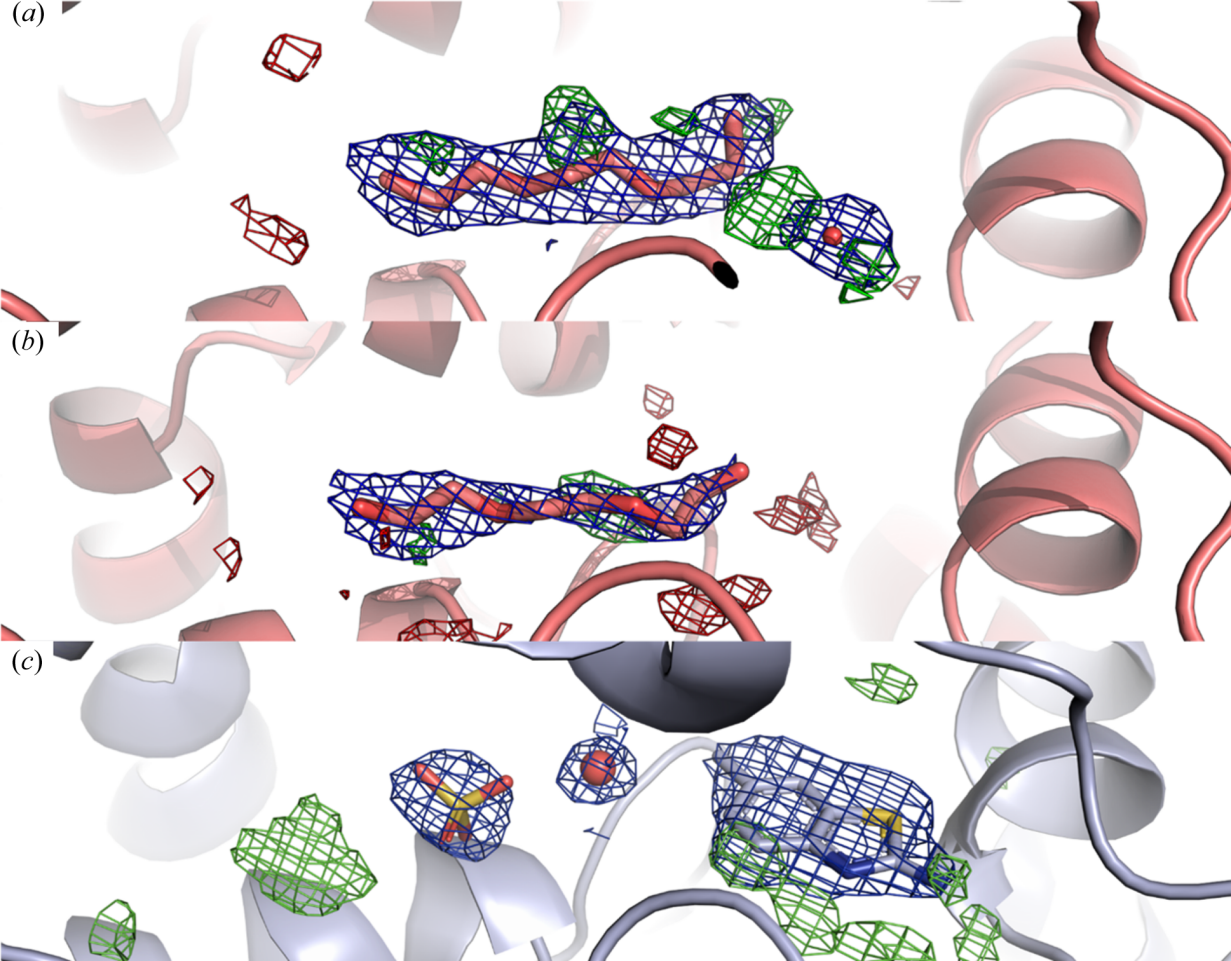
Temperature-dependent difference in compound binding mode. The RT structure from crystals soaked with compound 7 is compared with the RT apo structure and the cryo-T structure in complex with compound 7. (*a*) In the RT structure of sEH obtained from crystals soaked with compound 7, the active-site density is best fitted with a PEG fragment (PDB entry 8qwg). (*b*) RT apo structure with a PEG fragment bound in the active site (PDB entry 8qvm). (*c*) In the cryo-T structure from crystals soaked with compound 7 (PDB entry 5ai8; Öster *et al.*, 2015 ▸), the compound is bound as well as a sulfate ion and a water molecule. For each structure, the 2*F*_o_*F*_c_ electron density map is contoured at 1σ (blue) and the *F*_o_*F*_c_ electron density map at +3.5σ (green).

Finally, the structure generated from microcrystals soaked with compound 7 shows a large positive difference electron-density feature in the active site. However, the compound cannot be reasonably modelled and the density is instead well fitted with a PEG fragment (Fig. 5 ▸). To rule out the possibility that compound 7 binds at lower occupancy in the RT structure, a model with the PEG fragment at 50% occupancy and compound 7 in the adjacent position at 50% occupancy was tested but results in a very poor fit of the electron density (Fig. S4).

With the exception of compound 7, the structures agree well with the corresponding cryo-T structures. Interestingly, the RT structure solved from crystals soaked with compound 7 is distinctly different from that solved at cryo-T, where compound 7 is well fitted in the electron density at a position adjacent to the PEG position and not coordinated with the catalytic triad [Fig. 5 ▸(*c*)]. The fact that differences in ligand-binding features can be observed upon comparing structures solved at cryo-T and RT has been observed previously (Fischer *et al.*, 2015 ▸).

#### 3.3.1. Water molecules, *B* factors and loops

**Figure 6:**
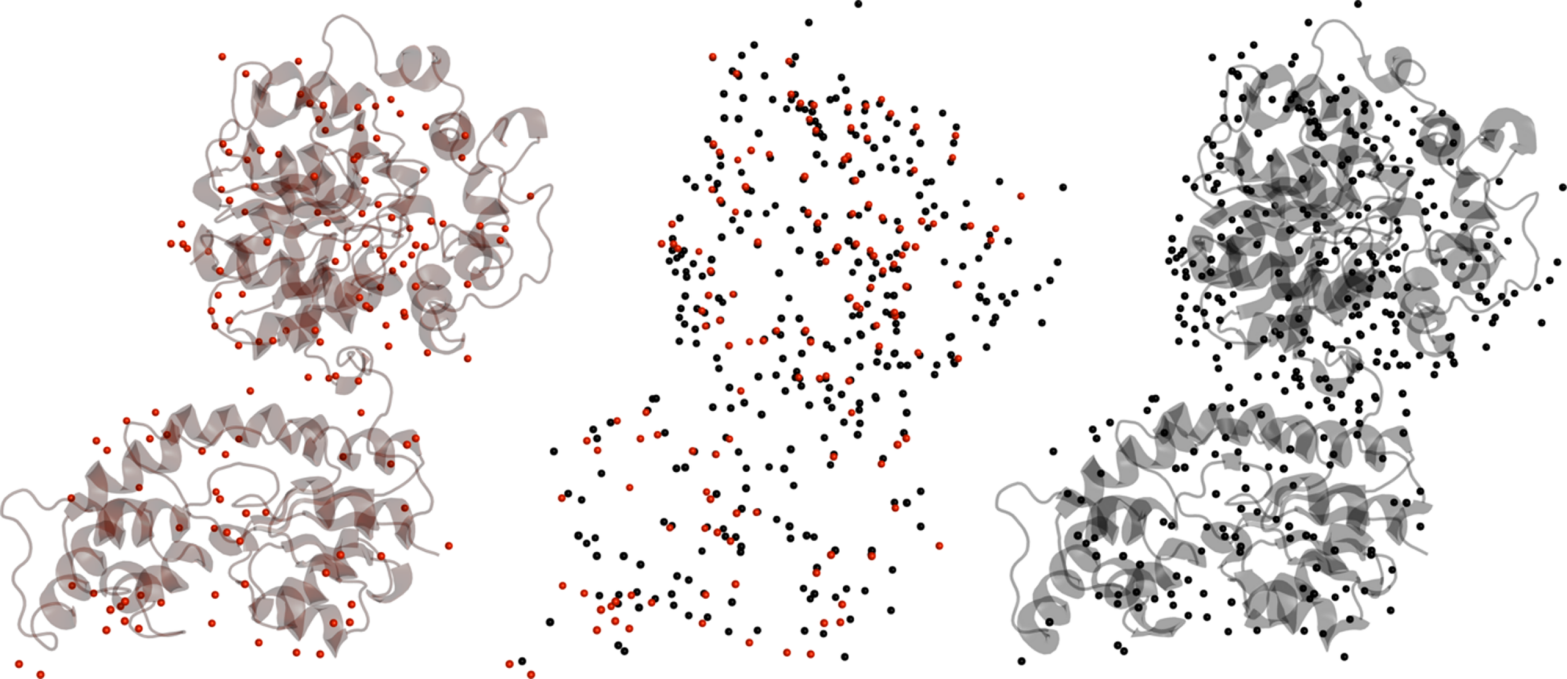
Comparison of water molecules modelled at RT and at cryo-T. (Left) RT sEH structure (in complex with compound 3, PDB entry 8qvk) with water molecules in red. (Right) Corresponding cryo-T structure (PDB entry 5ake) with water molecules in black. The central panel displays an overlay of the water molecules.

A significant difference between the sEH structures solved at RT and at cryo-T is the number of water molecules that can be modelled. The water content is on average more than two times higher in the structures solved using conventional cryo-crystallography (Fig. 6 ▸). This is likely due to the fact that the elevated temperature increases disorder of water molecules that are not highly coordinated, as has been observed by others (Nakasako, 1999 ▸, 2001 ▸). Despite this difference in water content, we find that many well ordered water molecules in the interior of the catalytic domain are also conserved in the SX structures.

**Figure 7:**
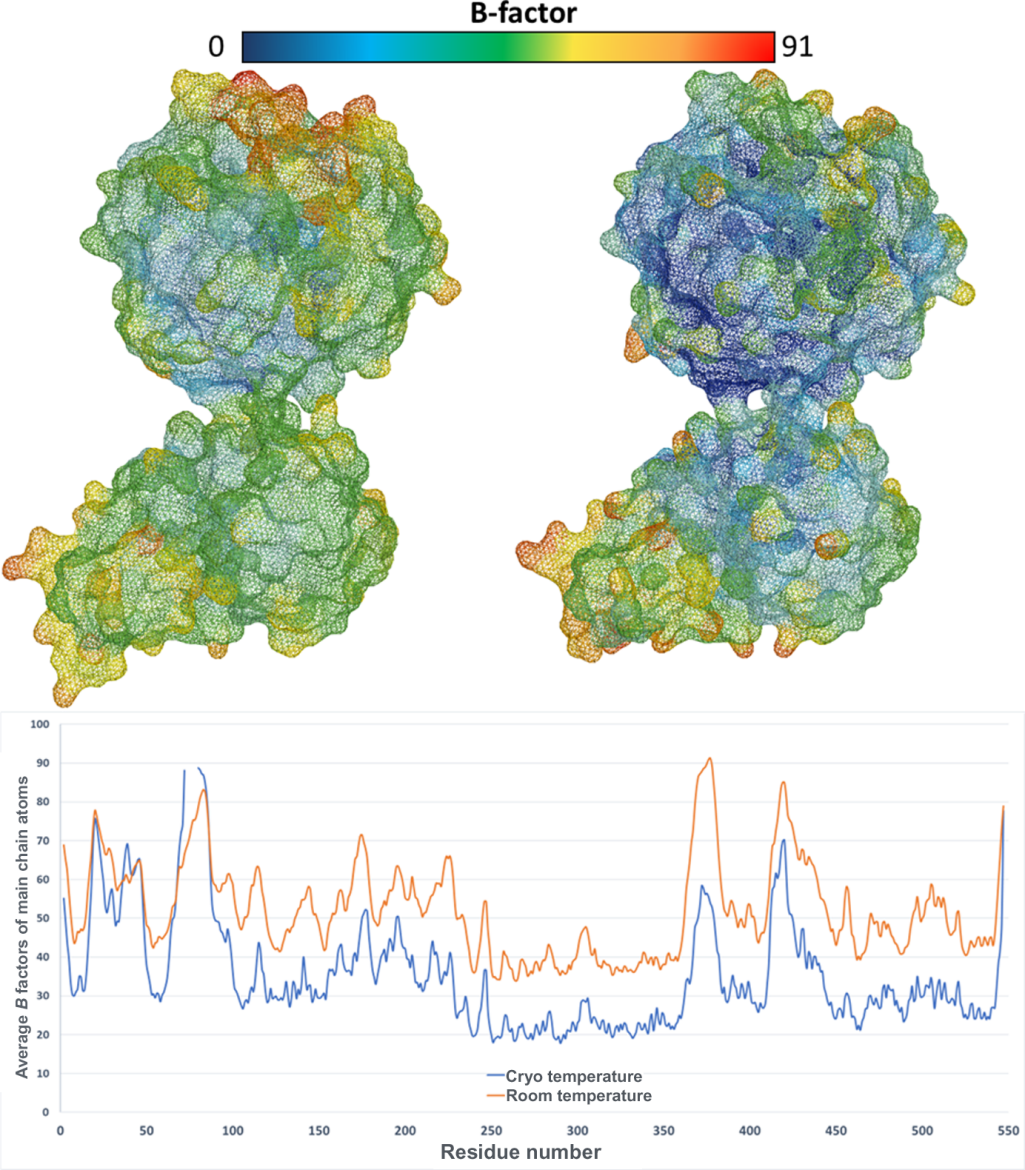
*B*-factor comparison. A *B*-factor comparison is shown for one RT structure (PDB entry 8qwg) and its cryo-T counterpart (PDB entry 5ai8). At the top, the RT (left-hand side) and cryo-T (right-hand side) sEH structures are displayed colored according to *B* factors. At the bottom, the average *B* factors of main chain atoms are plotted according to residue number (RT structure – orange, cryo-T structure – blue).

As expected, we observe higher *B* factors in the RT structures compared with the cryo-T structures. This difference can be attributed to several factors, including increased thermal motion and enhanced flexibility when the structure is not cryo-trapped. The crystal lattice of microcrystals may be more resilient to conformational changes, thus allowing the dynamics of the protein to be observed. Radiation damage can also be manifested in the form of high *B* factors, and care must be taken to stay below the dose limit of 5 × 10^5^ Gy when X-ray radiation induced damage is typically observed in data collected at RT (Schneps *et al.*, 2022 ▸). In our experiments, we calculate the radiation dose to ∼4.5 × 10^4^ Gy at each grid position of the chip (Bury *et al.*, 2018 ▸). In a recent study of a cryptochrome using fixed-target SX, it was noted that the refined *B* factors correlated well with those predicted from molecular-dynamics simulations (Schneps *et al.*, 2022 ▸), pointing towards the protein being in a fully solvated, functional state in the microcrystals. The structures of sEH display flexibility in some loop-regions at both temperatures, including those surrounding the active site. Interestingly, the *B* factors of the residues around the active site are significantly elevated in the RT structures relative to the cryo-T structures, suggesting a dynamic behaviour of the active site under ambient conditions that is masked in the frozen state (Fig. 7 ▸). This type of enhanced loop mobility of functionally important regions was reported recently in a lower-resolution SX study (Schneps *et al.*, 2022 ▸), where it was suggested that information gained from analysing dynamic behaviour could be useful to identify key structural elements in a protein.

**Figure 8:**
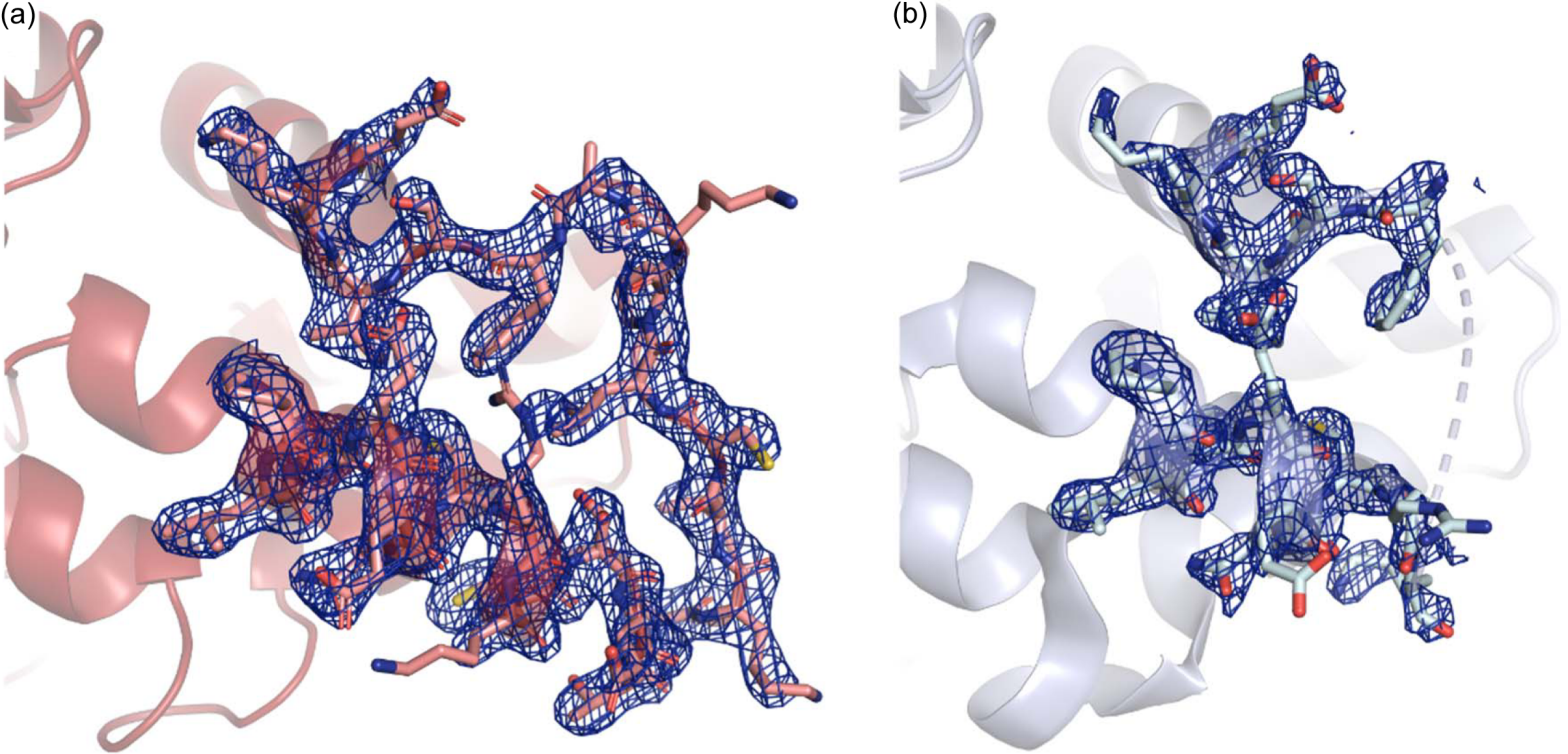
Ordering of loops. An example of a loop (Pro65 to Ala95) with a higher degree of order in the RT structures compared with in the cryo-T structures. (*a*) 2*F*_o_*F*_c_ electron density map associated with crystals soaked with compound 7 at RT (PDB entry 8qwg). (*b*) 2*F*_o_*F*_c_ electron density map associated with the corresponding cryo-T structure (PDB entry 5ai8). The 2*F*_o_*F*_c_ electron density maps are contoured at 1σ (blue).

One of the most pronounced differences related to temperature in the structures of sEH is that several of the loops are more well defined in the RT models. Thus, it seems that it is possible to obtain more information on structural regions that are typically less ordered by use of SX. In our SX structures, we can confidently model the loop composed of residues 65 to 95 in the N-terminal domain, whereas this loop is either not possible to model at all in the cryo-T structures or only associated with partial electron density (Fig. 8 ▸). Interestingly, this loop is close to the putative phosphatase region of sEH (Cronin *et al.*, 2003 ▸; Matsumoto *et al.*, 2019 ▸; Vázquez *et al.*, 2023 ▸). The fact that RT diffraction data can provide superior electron density maps of flexible regions has been noted previously in a study of a bacterial reaction centre using XFEL radiation (Dods *et al.*, 2017 ▸). The reason for this temperature effect may be related to the fact that flash-cooling induces an artificial thermal heterogeneity in the structure (Halle, 2004 ▸). In contrast, it was recently shown that lipid tails and detergent molecules were not as well resolved at higher temperature (Båth *et al.*, 2022 ▸).

### 3.4. Potential for optimization of the SX workflow

A serious limitation of how SX is used today is the time-consuming nature of the method. In the study presented here, the collection of each dataset typically took 1–2 h. We see several possible avenues to explore in order to achieve a more efficient workflow. In the case of our model system sEH, the speed of data collection could be greatly enhanced by collecting sufficient – but not excessive – amounts of data. To investigate how the quality of the electron density map is affected by reducing the number of images, we solved the structure of sEH in complex with compound 5 using a subset of the data containing ∼5000 indexed diffraction images (to be compared with the ∼65 000 indexed images used for the structure presented in Table 1 ▸). The structure resulting from the subset of the data is of similar resolution (2.2 Å based on the subset of data compared with 2.1 Å for the full dataset) and the ligand can be unambiguously placed in the active site electron density. Thus, it is clear that the very high redundancy, and thereby data collection time, of several of our datasets is not necessary and there is potential to reduce the data collection time by up to 5× using the current setup. Moreover, there are other sample delivery methods to be explored for efficient SX data collection (Zielinski *et al.*, 2022 ▸).

A future development that would significantly reduce the data collection time is to make the fixed targets compatible with the robotics systems at the beamlines so that mounting and dismounting of the chips could be achieved without manual handling.

Further, over the last couple of years, automatic data processing pipelines have been successfully established at many SX beamlines. This development offers rapid feedback regarding, for example, resolution during the experiment, and also reduces the time that has to be invested into each structure after the data are collected.

Another limitation that is currently associated with the method is the lack of reliable ways to ship or transport the microcrystals at RT without damaging them. On-chip crystallization has the potential to overcome this problem and may be a future path towards enabling remote collection also of SX data as it would circumvent the need for manual crystal handling during the experiment.

## 4. Conclusions

We have developed a robust workflow for efficient production of microcrystals for SX experiments. This involves the use of a hybrid crystallization approach to screen for suitable conditions using low amounts of protein. The method allows easy evaluation of crystallization trials and is readily scalable to larger volumes. Hybrid crystallization was successfully used to find microcrystallization conditions for the pharmaceutical target sEH, and based on this, larger amounts of microcrystals were produced by batch crystallization. We were able to solve RT high-quality structures of sEH in complex with six different ligands by SSX using a fixed-target setup. In comparison with previously published cryo-T structures of the same protein–ligand complexes, we found that the ligands generally bind in a similar fashion in the RT and the cryo-T structures. However, we observed one example of a temperature-dependent difference in the binding of a low-molecular-weight compound, information that may influence the chemical design in a drug discovery project. In addition, we observe differences in protein conformations and solvent molecules both in the vicinity of the active site and in the more remote regions. Thus, it may be beneficial to consider structural information at RT in a structure-based drug design project, in particular when working with weakly binding compounds.

Future studies will need to address challenges in working with poorly soluble ligands that cause precipitation, as well as issues with transporting and shipping crystals at RT. The most pressing challenge for SX from the perspective of pharmaceutical drug discovery is to tackle the slow turnover and labour-intense nature of this method. Indeed, there is potential to optimize the SX workflow by developing a more automated crystal handling process. If successful, SX could also become an attractive alternative for high-throughput drug discovery campaigns.

## Supplementary Material

Supporting figures and tables. DOI: 10.1107/S2052252524006134/jt5073sup1.pdf

PDB reference: RT structure of apo soluble epoxide hydrolase (sEH), 8qvm

PDB reference: RT structure of sEH with compound 1, 8qvh

PDB reference: RT structure of sEH with compound 2, 8qvf

PDB reference: RT structure of sEH with compound 3, 8qvk

PDB reference: RT structure of sEH with compound 4, 8qvg

PDB reference: RT structure of sEH with compound 5, 8qwi

PDB reference: RT structure of sEH with compound 6, 8qvl

PDB reference: RT structure of sEH with compound 7, 8qwg

## Figures and Tables

**Table 1:** Data collection, processing and refinement statistics

	Apo (8qvm)	Compound 1 (8qvh)	Compound 2 (8qvf)	Compound 3 (8qvk)	Compound 4 (8qvg)	Compound 5 (8qwi)	Compound 6 (8qvl)	Compound 7 PEG fragment (8qwg)
Data collection								
Diffraction source	MAX IV: BioMAX							
Wavelength (Å)	0.98							
Temperature (K)	293							
*a*, *b*, *c* (Å)	94.41 94.41, 247.57	95.41 95.41 244.29	94.33 94.33 246.92	94.43 94.43 246.89	93.87 93.87 246.37	93.95 93.95 246.69	94.39 94.39 247.45	94.4 94.4 246.96
α, β, γ (°)	90. 90. 120	90. 90. 120	90. 90. 120	90. 90. 120	90. 90. 120	90. 90. 120	90. 90. 120	90. 90. 120
Resolution range (Å)	44.11–2.00 (2.00–2.01)	49.11–2.24 (2.24–2.25)	44.44–2.4 (2.40–2.42)	49.45–2.1 (2.10–2.11)	38.6–2.20 (2.20–2.21)	49.15–2.12 (2.12–2.13)	38.81–2.14 (2.14–2.15)	44.09–2.20 (2.20–2.22)
Total no. of reflections	7597201	8738291	825452	3310882	16249553	77265832	122064109	1593253
No. of unique reflections	114534	61484	48145	72694	56223	69231	71073	60947
Completeness (outer shell) (%)	100 (100)	100 (100)	100 (100)	100 (100)	100 (100)	100 (100)	100 (100)	100 (100)
Redundancy (outer shell)	66 (49)	93 (57)	17 (11)	42 (28)	289 (189)	1116 (763)	1717 (1182)	26 (17)
〈*I*/σ(*I*)〉	3.66 (0.71)	6.22 (0.70)	5.58 (1.54)	5.96 (0.71)	6.20 (0.72)	9.42(1.62)	6.78 (0.79)	5.47 (0.80)
CC_1/2_	95.1 (45.9)	98.9 (43.7)	96.7 (57.7)	94.2 (30.0)	96.9 (48.3)	99.3 (77.0)	98.6 (42.5)	94.9 (32.9)
*R*_split_ (%)	18.3 (96.9)	13.4 (132.6)	14.7 (72.3)	10.6 (144.1)	12.8 (84.8)	7.5 (51)	10.1 (105.6)	16.9 (128.5)
Wilson *B* factor	42.7	44.5	66.1	54.7	65.8	43.4	46.6	53.2
Total No. of images	84855	43902	98275	41140	94426	94787	109977	32446
Indexed images	36503	27369	5432	21078	37162	65236	72003	6713

Structure refinement								
Resolution range (Å)	44.11–2.00	49.11 – 2.24	44.44–2.4	49.45–2.1	38.6–2.20	49.15–2.12	38.81–2-14	44.09–2.20
No. of reflections used in refinement (test)	45033 (2234)	32577 (1625)	26621 (1338)	39762 (2013)	33532 (1645)	37504 (1913)	36913 (1838)	33994 (1688)
*R*_work_/*R*_free_	19.70/24.60	18.73/22.80	18.58/22.28	18.82/22.83	21.61/25.49	17.46/20.19	18.86/22.87	19.97/23.29
Amino acids	547	547	547	547	547	547	547	547
Waters	297	184	98	158	197	209	159	143
Bonds (Å)	0.008	0.008	0.007	0.008	0.008	0.008	0.008	0.008
Angles (°)	0.94	0.95	0.94	0.94	0.96	0.93	0.94	0.95
Average *B* factors (Å^2^)	45	44.9	59.8	56.3	58.1	43.2	49.3	54.2
Average *B* factors compound(s) (Å^2^)		44	60.8	69.5	50.7	79	63.1	81.8
Average *B* factors solvent (Å^2^)	54.7	51.5	60.6	60.2	63.6	52.1	54.4	57.8
Ramachandran plot								
Favored regions (%)	97.4	96.70	95.9	97.2	96.40	96.9	97.2	97.4
Allowed (%)	2.42	2.93	3.91	2.61	3.23	2.91	2.42	2.04
Outliers (%)	0.18	0.37	0.19	0.19	0.37	0.19	0.38	0.56

## Data Availability

The atomic coordinates and structure factor files for the sEH structure are available under PDB entries 8qvm, 8qvl, 8qvk, 8qwi, 8qwg, 8qvh, 8qvg and 8qvf at https://www.pdb.org.

## References

[bb2] Agirre, J., Atanasova, M., Bagdonas, H., Ballard, C. B., Baslé, A., Beilsten-Edmands, J., Borges, R. J., Brown, D. G., Burgos-Mármol, J. J., Berrisford, J. M., Bond, P. S., Caballero, I., Catapano, L., Chojnowski, G., Cook, A. G., Cowtan, K. D., Croll, T. I., Debreczeni, J. É., Devenish, N. E., Dodson, E. J., Drevon, T. R., Emsley, P., Evans, G., Evans, P. R., Fando, M., Foadi, J., Fuentes-Montero, L., Garman, E. F., Gerstel, M., Gildea, R. J., Hatti, K., Hekkelman, M. L., Heuser, P., Hoh, S. W., Hough, M. A., Jenkins, H. T., Jiménez, E., Joosten, R. P., Keegan, R. M., Keep, N., Krissinel, E. B., Kolenko, P., Kovalevskiy, O., Lamzin, V. S., Lawson, D. M., Lebedev, A. A., Leslie, A. G. W., Lohkamp, B., Long, F., Malý, M., McCoy, A. J., McNicholas, S. J., Medina, A., Millán, C., Murray, J. W., Murshudov, G. N., Nicholls, R. A., Noble, M. E. M., Oeffner, R., Pannu, N. S., Parkhurst, J. M., Pearce, N., Pereira, J., Perrakis, A., Powell, H. R., Read, R. J., Rigden, D. J., Rochira, W., Sammito, M., Sánchez Rodríguez, F., Sheldrick, G. M., Shelley, K. L., Simkovic, F., Simpkin, A. J., Skubak, P., Sobolev, E., Steiner, R. A., Stevenson, K., Tews, I., Thomas, J. M. H., Thorn, A., Valls, J. T., Uski, V., Usón, I., Vagin, A., Velankar, S., Vollmar, M., Walden, H., Waterman, D., Wilson, K. S., Winn, M. D., Winter, G., Wojdyr, M. & Yamashita, K. (2023). *Acta Cryst.* D**79**, 449–461.

[bb3] Båth, P., Banacore, A., Börjesson, P., Bosman, R., Wickstrand, C., Safari, C., Dods, R., Ghosh, S., Dahl, P., Ortolani, G., Björg Ulfarsdottir, T., Hammarin, G., García Bonete, M.-J., Vallejos, A., Ostojić, L., Edlund, P., Linse, J.-B., Andersson, R., Nango, E., Owada, S., Tanaka, R., Tono, K., Joti, Y., Nureki, O., Luo, F., James, D., Nass, K., Johnson, P. J. M., Knopp, G., Ozerov, D., Cirelli, C., Milne, C., Iwata, S., Brändén, G. & Neutze, R. (2022). *Acta Cryst.* D**78**, 698–708. 10.1107/S2059798322004144 PMC9159286 35647917

[bb4] Beale, J. H., Bolton, R., Marshall, S. A., Beale, E. V., Carr, S. B., Ebrahim, A., Moreno-Chicano, T., Hough, M. A., Worrall, J. A. R., Tews, I. & Owen, R. L. (2019). *J. Appl. Cryst.* **52**, 1385–1396. 10.1107/S1600576719013517 PMC6878878 31798361

[bb5] Boudes, M., Garriga, D. & Coulibaly, F. (2017). *J. Vis. Exp.* **125**, 55793. 10.3791/55793 PMC5612586 28784967

[bb6] Boutet, S., Lomb, L., Williams, G. J., Barends, T. R. M., Aquila, A., Doak, R. B., Weierstall, U., DePonte, D. P., Steinbrener, J., Shoeman, R. L., Messerschmidt, M., Barty, A., White, T. A., Kassemeyer, S., Kirian, R. A., Seibert, M. M., Montanez, P. A., Kenney, C., Herbst, R., Hart, P., Pines, J., Haller, G., Gruner, S. M., Philipp, H. T., Tate, M. W., Hromalik, M., Koerner, L. J., van Bakel, N., Morse, J., Ghonsalves, W., Arnlund, D., Bogan, M. J., Caleman, C., Fromme, R., Hampton, C. Y., Hunter, M. S., Johansson, L. C., Katona, G., Kupitz, C., Liang, M., Martin, A. V., Nass, K., Redecke, L., Stellato, F., Timneanu, N., Wang, D., Zatsepin, N. A., Schafer, D., Defever, J., Neutze, R., Fromme, P., Spence, J. C. H., Chapman, H. N. & Schlichting, I. (2012). *Science*, **337**, 362–364.

[bb7] Brändén, G. & Neutze, R. (2021). *Science*, **373**, eaba0954. 10.1126/science.aba0954 34446579

[bb8] Bricogne, G., Blanc, E., Brandl, M., Flensburg, C., Keller, P., Paciorek, W., Roversi, P., Sharff, A., Smart, O. S., Vonrhein, C. & Womack (2017). *BUSTER*. Global Phasing Ltd, Cambridge, United Kingdom.

[bb9] Bury, C. S., Brooks–Bartlett, J. C., Walsh, S. P. & Garman, E. F. (2018). *Protein Sci.* **27**, 217–228. 10.1002/pro.3302 PMC5734275 28921782

[bb10] Caramello, N. & Royant, A. (2024). *Acta Cryst.* D**80**, 60–79. 10.1107/S2059798323011002 PMC10836399 38265875

[bb11] Carrillo, M., Mason, T., Karpik, A., Martiel, I., Kepa, M., McAuley, K., Beale, J. & Padeste, C. (2023). *IUCrJ*, **10**, 678–693. 10.1107/S2052252523007595 PMC10619457 37727961

[bb12] Chapman, H. N., Fromme, P., Barty, A., White, T. A., Kirian, R. A., Aquila, A., Hunter, M. S., Schulz, J., DePonte, D. P., Weierstall, U., Doak, R. B., Maia, F. R. N. C., Martin, A. V., Schlichting, I., Lomb, L., Coppola, N., Shoeman, R. L., Epp, S. W., Hartmann, R., Rolles, D., Rudenko, A., Foucar, L., Kimmel, N., Weidenspointner, G., Holl, P., Liang, M., Barthelmess, M., Caleman, C., Boutet, S., Bogan, M. J., Krzywinski, J., Bostedt, C., Bajt, S., Gumprecht, L., Rudek, B., Erk, B., Schmidt, C., Hömke, A., Reich, C., Pietschner, D., Strüder, L., Hauser, G., Gorke, H., Ullrich, J., Herrmann, S., Schaller, G., Schopper, F., Soltau, H., Kühnel, K.-U., Messerschmidt, M., Bozek, J. D., Hau-Riege, S. P., Frank, M., Hampton, C. Y., Sierra, R. G., Starodub, D., Williams, G. J., Hajdu, J., Timneanu, N., Seibert, M. M., Andreasson, J., Rocker, A., Jönsson, O., Svenda, M., Stern, S., Nass, K., Andritschke, R., Schröter, C.-D., Krasniqi, F., Bott, M., Schmidt, K. E., Wang, X., Grotjohann, I., Holton, J. M., Barends, T. R. M., Neutze, R., Marchesini, S., Fromme, R., Schorb, S., Rupp, D., Adolph, M., Gorkhover, T., Andersson, I., Hirsemann, H., Potdevin, G., Graafsma, H., Nilsson, B. & Spence, J. C. H. (2011). *Nature*, **470**, 73–77.

[bb13] Chiamvimonvat, N., Ho, C.-M., Tsai, H.-J. & Hammock, B. D. (2007). *J. Cardiovasc. Pharmacol.* **50**, 225–237. 10.1097/FJC.0b013e3181506445 17878749

[bb14] Cronin, A., Mowbray, S., Dürk, H., Homburg, S., Fleming, I., Fisslthaler, B., Oesch, F. & Arand, M. (2003). *Proc. Natl Acad. Sci. USA*, **100**, 1552–1557. 10.1073/pnas.0437829100 PMC149870 12574508

[bb15] Dods, R., Båth, P., Arnlund, D., Beyerlein, K. R., Nelson, G., Liang, M., Harimoorthy, R., Berntsen, P., Malmerberg, E., Johansson, L., Andersson, R., Bosman, R., Carbajo, S., Claesson, E., Conrad, C. E., Dahl, P., Hammarin, G., Hunter, M. S., Li, C., Lisova, S., Milathianaki, D., Robinson, J., Safari, C., Sharma, A., Williams, G., Wickstrand, C., Yefanov, O., Davidsson, J., DePonte, D. P., Barty, A., Brändén, G. & Neutze, R. (2017). *Structure*, **25**, 1461–1468.e2. 10.1016/j.str.2017.07.002 28781082

[bb16] Ebrahim, A., Riley, B. T., Kumaran, D., Andi, B., Fuchs, M. R., McSweeney, S. & Keedy, D. A. (2022). *IUCrJ*, **9**, 682–694. 10.1107/S2052252522007497 PMC9438506 36071812

[bb17] Emsley, P. & Cowtan, K. (2004). *Acta Cryst.* D**60**, 2126–2132. 10.1107/S0907444904019158 15572765

[bb18] Fenwick, R. B., van den Bedem, H., Fraser, J. S. & Wright, P. E. (2014). *Proc. Natl Acad. Sci.* **111**, E445–E454. 10.1073/pnas.1323440111 PMC3910589 24474795

[bb19] Fischer, M., Shoichet, B. K. & Fraser, J. S. (2015). *ChemBioChem*, **16**, 1560–1564. 10.1002/cbic.201500196 PMC4539595 26032594

[bb20] Gati, C., Bourenkov, G., Klinge, M., Rehders, D., Stellato, F., Oberthür, D., Yefanov, O., Sommer, B. P., Mogk, S., Duszenko, M., Betzel, C., Schneider, T. R., Chapman, H. N. & Redecke, L. (2014). *IUCrJ*, **1**, 87–94. 10.1107/S2052252513033939 PMC4062088 25075324

[bb21] Ghosh, S., Zorić, D., Dahl, P., Bjelčić, M., Johannesson, J., Sandelin, E., Borjesson, P., Björling, A., Banacore, A., Edlund, P., Aurelius, O., Milas, M., Nan, J., Shilova, A., Gonzalez, A., Mueller, U., Brändén, G. & Neutze, R. (2023). *J. Appl. Cryst.* **56**, 449–460. 10.1107/S1600576723001036 PMC10077854 37032973

[bb22] Guaita, M., Watters, S. C. & Loerch, S. (2022). *Curr. Opin. Struct. Biol.* **77**, 102484. 10.1016/j.sbi.2022.102484 PMC10266358 36323134

[bb23] Halle, B. (2004). *Proc. Natl Acad. Sci. USA*, **101**, 4793–4798. 10.1073/pnas.0308315101 PMC387327 15051877

[bb24] Hashimoto, K. (2019). *Front. Pharmacol.* **10**, https://doi.org/10.3389/fphar.2019.00036.

[bb25] Imig, J. D. & Hammock, B. D. (2009). *Nat. Rev. Drug Discov.* **8**, 794–805. 10.1038/nrd2875 PMC3021468 19794443

[bb26] Keedy, D. A., Hill, Z. B., Biel, J. T., Kang, E., Rettenmaier, T. J., Brandão-Neto, J., Pearce, N. M., von Delft, F., Wells, J. A. & Fraser, J. S. (2018). *eLife*, **7**, e36307. 10.7554/eLife.36307 PMC6039181 29877794

[bb27] Martin-Garcia, J. M. (2021). *Crystals*, **11**, 521.

[bb28] Matsumoto, N., Kataoka, M., Hirosaki, H., Morisseau, C., Hammock, B. D., Suzuki, E. & Hasumi, K. (2019). *Biochem. Biophys. Res. Commun.* **515**, 248–253. 10.1016/j.bbrc.2019.05.088 PMC7395850 31146915

[bb29] Maveyraud, L. & Mourey, L. (2020). *Molecules*, **25**, 1030. 10.3390/molecules25051030 PMC7179213 32106588

[bb30] Mehrabi, P., Müller-Werkmeister, H. M., Leimkohl, J.-P., Schikora, H., Ninkovic, J., Krivokuca, S., Andriček, L., Epp, S. W., Sherrell, D., Owen, R. L., Pearson, A. R., Tellkamp, F., Schulz, E. C. & Miller, R. J. D. (2020). *J. Synchrotron Rad.* **27**, 360–370. 10.1107/S1600577520000685 PMC7064102 32153274

[bb31] Milano, S. K., Huang, Q., Nguyen, T.-T. T., Ramachandran, S., Finke, A., Kriksunov, I., Schuller, D. J., Szebenyi, D. M., Arenholz, E., McDermott, L. A., Sukumar, N., Cerione, R. A. & Katt, W. P. (2021). *J. Biol. Chem.* **298**, 101535. 10.1016/j.jbc.2021.101535 PMC8784640 34954143

[bb32] Nakasako, M. (1999). *J. Mol. Biol.* **289**, 547–564. 10.1006/jmbi.1999.2795 10356328

[bb33] Nakasako, M. (2001). *Cell. Mol. Biol.* **47**, 767–790. 11728092

[bb34] Newman, J. W., Morisseau, C., Harris, T. R. & Hammock, B. D. (2003). *Proc. Natl Acad. Sci. USA*, **100**, 1558–1563. 10.1073/pnas.0437724100 PMC149871 12574510

[bb35] Öster, L., Tapani, S., Xue, Y. & Käck, H. (2015). *Drug Discovery Today*, **20**, 1104–1111. 10.1016/j.drudis.2015.04.005 25931264

[bb36] Owen, R. L., Axford, D., Sherrell, D. A., Kuo, A., Ernst, O. P., Schulz, E. C., Miller, R. J. D. & Mueller-Werkmeister, H. M. (2017). *Acta Cryst.* D**73**, 373–378. 10.1107/S2059798317002996 PMC5379936 28375148

[bb37] Roedig, P., Duman, R., Sanchez-Weatherby, J., Vartiainen, I., Burkhardt, A., Warmer, M., David, C., Wagner, A. & Meents, A. (2016). *J. Appl. Cryst.* **49**, 968–975. 10.1107/S1600576716006348 PMC4886986 27275143

[bb38] Roedig, P., Ginn, H. M., Pakendorf, T., Sutton, G., Harlos, K., Walter, T. S., Meyer, J., Fischer, P., Duman, R., Vartiainen, I., Reime, B., Warmer, M., Brewster, A. S., Young, I. D., Michels-Clark, T., Sauter, N. K., Kotecha, A., Kelly, J., Rowlands, D. J., Sikorsky, M., Nelson, S., Damiani, D. S., Alonso-Mori, R., Ren, J., Fry, E. E., David, C., Stuart, D. I., Wagner, A. & Meents, A. (2017). *Nat. Methods*, **14**, 805–810. 10.1038/nmeth.4335 PMC5588887 28628129

[bb39] Schiebel, J., Krimmer, S. G., Röwer, K., Knörlein, A., Wang, X., Park, A. Y., Stieler, M., Ehrmann, F. R., Fu, K., Radeva, N., Krug, M., Huschmann, F. U., Glöckner, S., Weiss, M. S., Mueller, U., Klebe, G. & Heine, A. (2016). *Structure*, **24**, 1398–1409. 10.1016/j.str.2016.06.010 27452405

[bb40] Schneps, C. M., Ganguly, A. & Crane, B. R. (2022). *Acta Cryst.* D**78**, 975–985. 10.1107/S2059798322007008 PMC9344480 35916222

[bb41] Schrödinger, LLC (2015). *The pyMOL Molecular Graphics System*, Version 1.8.

[bb42] Shen, H. C. & Hammock, B. D. (2012). *J. Med. Chem.* **55**, 1789–1808. 10.1021/jm201468j PMC3420824 22168898

[bb43] Shilova, A., Lebrette, H., Aurelius, O., Nan, J., Welin, M., Kovacic, R., Ghosh, S., Safari, C., Friel, R. J., Milas, M., Matej, Z., Högbom, M., Brändén, G., Kloos, M., Shoeman, R. L., Doak, B., Ursby, T., Håkansson, M., Logan, D. T. & Mueller, U. (2020). *J. Synchrotron Rad.* **27**, 1095–1102. 10.1107/S1600577520008735 PMC7467353 32876583

[bb44] Shoeman, R. L., Hartmann, E. & Schlichting, I. (2023). *Nat. Protoc.* **18**, 854–882. 10.1038/s41596-022-00777-5 36451055

[bb45] Skaist Mehlman, T., Biel, J. T., Azeem, S. M., Nelson, E. R., Hossain, S., Dunnett, L., Paterson, N. G., Douangamath, A., Talon, R., Axford, D., Orins, H., von Delft, F. & Keedy, D. A. (2023). *eLife*, **12**, e84632. 10.7554/eLife.84632 PMC9991056 36881464

[bb46] Smart, O. S., Womack, T. O., Sharff, A., Flensburg, C., Keller, P., Paciorek, W., Vonrhein, C., Bricogne, G., Sharff, A., Flensburg, C., Keller, P., Paciorek, W., Vonrhein, C. & Bricogne, G (2011). *Grade*. Global Phasing Ltd, Cambridge, United Kingdom.

[bb47] Sun, C.-P., Zhou, J.-J., Yu, Z.-L., Huo, X.-K., Zhang, J., Morisseau, C., Hammock, B. D. & Ma, X.-C. (2022). *Proc. Natl Acad. Sci.* **119**, e2118818119. 10.1073/pnas.2118818119 PMC8892522 35217618

[bb48] Vakili, M., Han, H., Schmidt, C., Wrona, A., Kloos, M., de Diego, I., Dörner, K., Geng, T., Kim, C., Koua, F. H. M., Melo, D. V. M., Rappas, M., Round, A., Round, E., Sikorski, M., Valerio, J., Zhou, T., Lorenzen, K. & Schulz, J. (2023). *J. Appl. Cryst.* **56**, 1038–1045. 10.1107/S1600576723004405 PMC10405586 37555221

[bb49] Vázquez, J., Ginex, T., Herrero, A., Morisseau, C., Hammock, B. D. & Luque, F. J. (2023). *J. Chem. Inf. Model.* **63**, 3209–3225. 10.1021/acs.jcim.3c00301 PMC10207366 37141492

[bb50] Weierstall, U., James, D., Wang, C., White, T. A., Wang, D., Liu, W., Spence, J. C. H., Bruce Doak, R., Nelson, G., Fromme, P., Fromme, R., Grotjohann, I., Kupitz, C., Zatsepin, N. A., Liu, H., Basu, S., Wacker, D., Won Han, G., Katritch, V., Boutet, S., Messerschmidt, M., Williams, G. J., Koglin, J. E., Marvin Seibert, M., Klinker, M., Gati, C., Shoeman, R. L., Barty, A., Chapman, H. N., Kirian, R. A., Beyerlein, K. R., Stevens, R. C., Li, D., Shah, S. T. A., Howe, N., Caffrey, M. & Cherezov, V. (2014). *Nat. Commun.* **5**, 3309. 10.1038/ncomms4309 PMC4061911 24525480

[bb51] White, T. A. (2019). *Acta Cryst.* D**75**, 219–233. 10.1107/S205979831801238X PMC6400257 30821710

[bb52] White, T. A., Kirian, R. A., Martin, A. V., Aquila, A., Nass, K., Barty, A. & Chapman, H. N. (2012). *J. Appl. Cryst.* **45**, 335–341.

[bb900] Wlodek, S., Skillman, A. G. & Nicholls, A. (2006). *Acta Cryst.* D**62**, 741–749. 10.1107/S0907444906016076 16790930

[bb53] Zielinski, K. A., Prester, A., Andaleeb, H., Bui, S., Yefanov, O., Catapano, L., Henkel, A., Wiedorn, M. O., Lorbeer, O., Crosas, E., Meyer, J., Mariani, V., Domaracky, M., White, T. A., Fleckenstein, H., Sarrou, I., Werner, N., Betzel, C., Rohde, H., Aepfelbacher, M., Chapman, H. N., Perbandt, M., Steiner, R. A. & Oberthuer, D. (2022). *IUCrJ*, **9**, 778–791. 10.1107/S2052252522010193 PMC9634612 36381150

