# Supplementary material for: Exploring serial crystallography for drug discovery
Source: IUCrJ. 2024 Jul 29;11(Pt 5):831–42. doi: 10.1107/S2052252524006134 (PMC11364032; doi:10.1107/S2052252524006134)
Supplement: Supplementary file 1 [file m-11-00831-sup1.pdf]

IUCrJ

Volume 11 (2024)

Supporting information for article:

Exploring serial crystallography for drug discovery

A. Dunge, C. Phan, O. Uwangue, M. Bjelcic, J. Gunnarsson, G. Wehlander, H. Käck and G. Brändén

Supplementary Materials for

Exploring serial crystallography for drug discovery

A. Dunge *et al.*

*Corresponding authors. Email: gisela.branden@gu.se, helena.kack@astrazeneca.com

This PDF file includes:

Table S1
Table S2
Fig. S1
Fig. S2
Fig. S3
Fig. S4

Table S1

Crystallization

**Method**	Vapor-diffusion	Hybrid-crystallization	Batch-crystallization
**Plate type**	24-well sitting drop plate	24-well sitting drop plate	0.5 mL Eppendorf Tubes
**Temperature (°C)**	20	20	20
**Protein concentration (mg/mL)**	16	10-20	14
**Protein buffer**	20 mM Tris-Cl, pH 8.0, 100 mM NaCl, 10% glycerol and 1 mM TCEP	20 mM Tris-Cl, pH 8.0, 100 mM NaCl, 10% glycerol and 1 mM TCEP	20 mM Tris-Cl, pH 8.0, 100 mM NaCl, 10% glycerol and 1 mM TCEP
**Precipitant solution**	32-38% PEG 3350, 0.1 M $LiSO_4$, 0.1 M Tris pH 8.5.	16-43% PEG 3350, 0.05-0.5 M $LiSO_4$, 0.05-0.5 M Tris pH 8.5.	34% PEG 3350, 0.1 M $LiSO_4$, 0.1 M Tris pH 8.5.
**Volume and ratio of drop (p:w)**	Total volume 5-10 µL, 1:1	Total volume 10 µL, 1:2 - 1:6	Total volume 50-100 µL, 1:4
**Crystallization reservoir**	500 µL	500 µL	-
**Seeding**	yes, streak seed	yes, 5-15% seed	yes, 10% seed (diluted 1:32-1:64)

Table S2

Compounds

Name (PDB id)	IUPAC Name	MW (Da)	IC50 (µM)	Solubility (µM)	Heavy atoms	LogD pH7.4 (calculated)
**Compound 1 (8QVH)**	4-[(trans-4-{[(3s,5s,7s)-tricyclo[3.3.1.1~3,7~]dec-1-ylcarbamoyl]amino}cyclohexyl)oxy]benzoic acid	413	0.005	390	30	2.0
**Compound 2 (8QVF)**	1-(1-adamantyl)-3-(1-methylsulfonylpiperidin-4-yl)urea	356	0.006	64	24	2.3
**Compound 3 (8QVK)**	N-(5,5-dioxodibenzothiophen-2-yl)-4,4-difluoro-piperidine-1-carboxamide	378	0.015	5	26	2.4
**Compound 4 (8QVG)**	N-(3,3-DIPHENYLPROPYL)PYRROLIDINE-1-CARBOXAMIDE	308	<0.086	97	23	3.6
**Compound 5 (8QWI)**	2-(1H-BENZIMIDAZOL-2-YLSULFANYL)ETHANOL	194	<1.37	>1000	13	1.3
**Compound 6 (8QVL)**	2-[(5-BROMO-2-PYRIDYL)-METHYL-AMINO]ETHANOL	231	332	0.9	12	2.1
**Compound 7 (8QWG)**	6-methyl-1,3-benzothiazol-2-amine	164	126	436	11	2.4

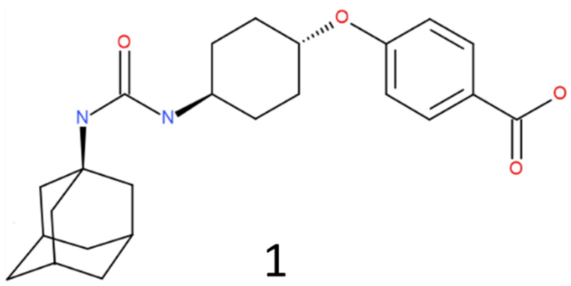

1

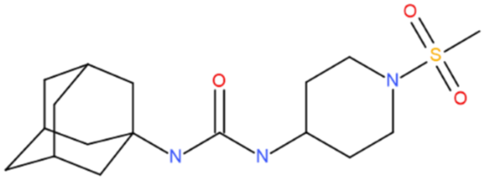

2

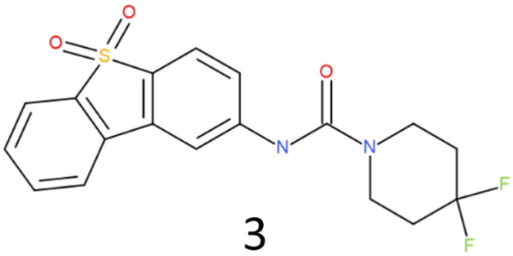

3

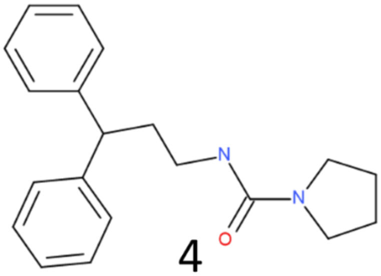

4

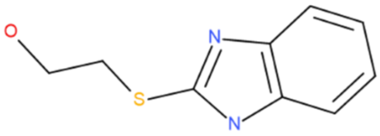

5

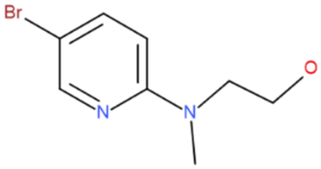

6

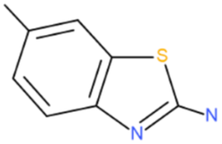

7

Figure S1. The 2D diagrams of compounds 1-7.

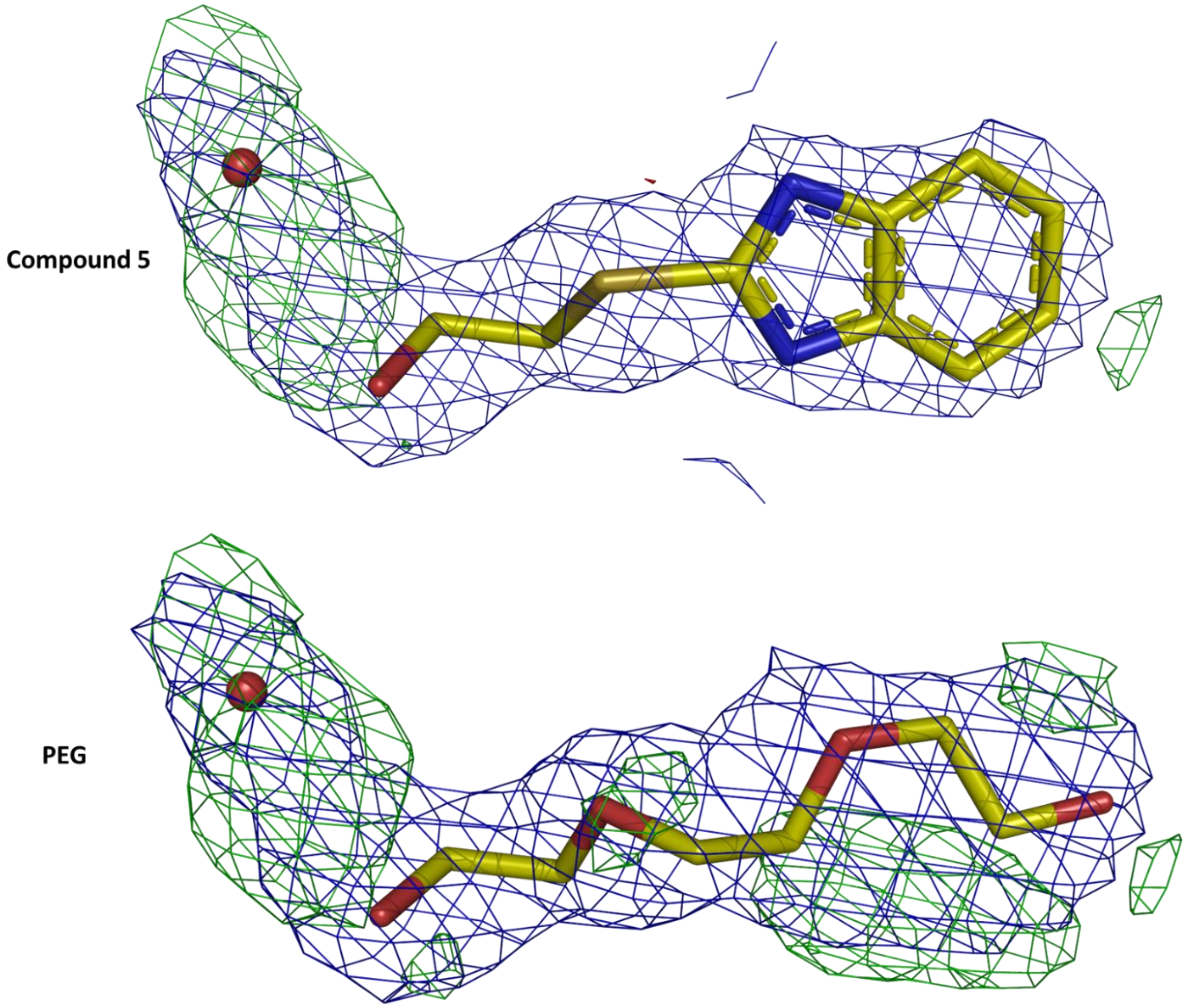

Figure S2. The room-temperature structure from crystals soaked with compound 5. The top panel shows the electron-density map associated with the room-temperature structure of sEH from crystals soaked with compound 5 refined with 100 % occupancy of the compound. The bottom panel shows the structure refined with a PEG fragment at an occupancy of 100 %. The resulting 2FoFc electron-density map is contoured at 1 σ (blue) and the FoFc electron-density map at +3.0 σ (green).

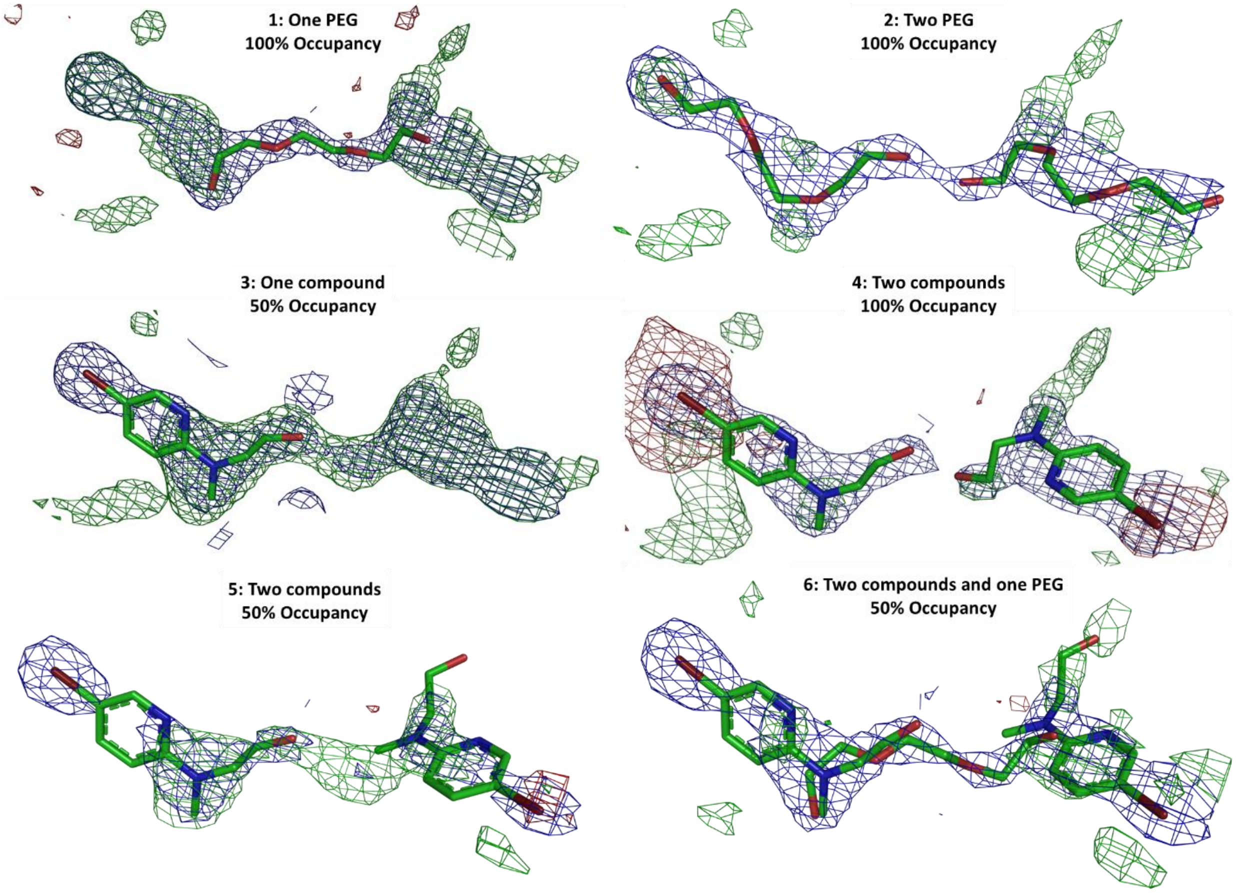

Figure S3. The room-temperature structure from crystals soaked with compound 6. The panels display the electron-density maps associated with the room-temperature structure of sEH from crystals soaked with compound 6 refined using different alternatives as active-site ligands. Panel 1: one PEG fragment in the position of the apo structure at 100 % occupancy. Panel 2: two PEG fragments at 100 % occupancy. Panel 3: one copy of the compound (associated with a strong Br signal) at an occupancy of 50 %. Panel 4: two copies of the compound at 100 % occupancy each. Panel 5: two copies of the compound with 50 % occupancy each. Panel 6: two copies of the compound and one PEG fragment at 50 % occupancy each. This gives the best fit to the data. The resulting 2FoFc electron-density map is contoured at 1 σ (blue) and the FoFc electron density map at +3.0 σ (green) and -3.0 σ (red).

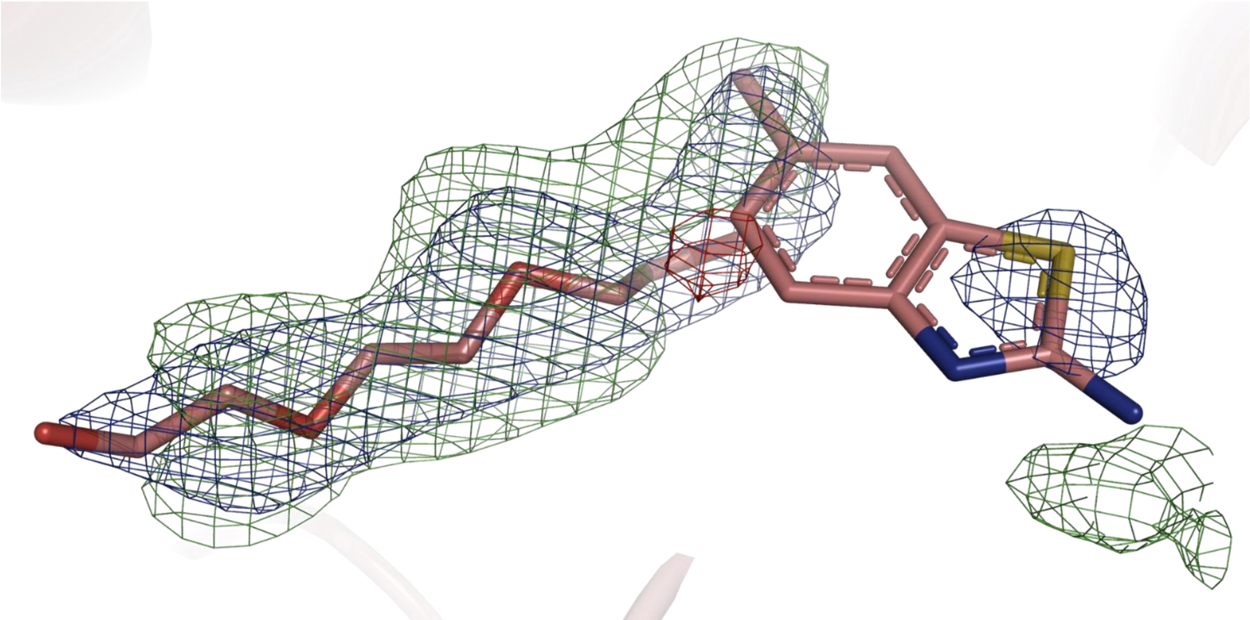

Figure S4. The room-temperature structure from crystals soaked with compound 7. The electron-density map associated with the room-temperature structure of sEH from crystals soaked with compound 7 refined with 50 % occupancy of the PEG fragment and 50 % occupancy of compound 7. A test with 30 % occupancy of compound 7 gave similar results. The resulting 2FoFc electron-density map is contoured at 1 σ (blue) and the FoFc electron-density map at +3.0 σ (green) and -3.0 σ (red).
